# Functional outcomes in post Covid-19 patients with persistent dyspnea: multidisciplinary approach

**DOI:** 10.1007/s10554-023-02819-9

**Published:** 2023-03-06

**Authors:** Rehab M. Hamdy, Ola Hassan Abdelaziz, Hager Elsayed Shamsseldain, Heba H. Eltrawy

**Affiliations:** 1 Department of cardiology, Faculty of Medicine (for Girls), Al-Azhar University, Cairo, Egypt; 2 Department of Chest, Faculty of Medicine (for Girls), Al-Azhar University, Cairo, Egypt

**Keywords:** Post Covid-19 syndrome, Persistent dyspnea, 2D-STE-LA strain

## Abstract

Background: Post-acute sequelae of SARS-CoV-2 (PASC) have emerged as a major health issue in patients who have previously been infected with Covid-19 virus. Purpose: we aimed at the assessment of functional outcomes in post Covid-19 patients with persistent dyspnea using a multidisciplinary approach including clinical assessment, laboratory investigations, exercise ECG, and different echo-Doppler modalities, including left atrial functions. Methods: The current observational randomized controlled study conducted on 60- patients one month after recovery from Covid-19 infection presented with persistent dyspnea compared to 30 healthy volunteers. All participants were subjected to evaluation of dyspnea by different scores, laboratory investigations, stress ECG, and echo-Doppler examination to measure LV dimensions, volumes, systolic and diastolic functions by M-mode, 2D, and tissue Doppler imaging in addition to 2-D speckle tacking LA strain. Results: Post Covid-19 patients had persistent elevation of inflammatory markers, low functional capacity (evidenced by a higher NYHA class, m MRC score, PCFS scale) and decreased METs by stress ECG compared to control group. Post Covid-19 patients showed LV diastolic dysfunction and impairment of 2D-STE LA functions compared to control group. We found negative correlations between LA strain with NYHA class, mMRC scale, LAVI, ESR and CRP; meanwhile, there were significant positive correlations between LA strain with exercise duration and METs. Conclusion: post Covid patients presented with persistent dyspnea demonstrated low functional capacity evidenced by different scores and stress ECG. Moreover, patients with post Covid syndrome showed elevated inflammatory biomarkers, LV diastolic dysfunction in addition to impaired LA strain functions. Impairment of LA strain was closely correlated to different functional scores, inflammatory biomarkers, exercise duration, and METs suggesting that these could to be the possible etiologies for the persistence of post Covid symptoms.

## Introduction

Severe acute respiratory syndrome coronavirus-2 (SARS-CoV-2) infection has been confirmed in millions of people around the world in the last few years [1] resulting in hospitalization in thousands of cases. Only 13% of previously hospitalized Covid-19 patients were fully free of any Covid-19-related symptom 2 months after the beginning of the first, while, 32% had one or two symptoms, and 55% had three or more [2].

Next to hospitalized patients with “severe” Covid-19, millions of people have most probably been infected with SARS-CoV-2 without formal Covid-19 testing and/or medical treatment in the hospital [3], and [4]. These patients were classified as having “mild” Covid-19 as they only required home care and the infection was expected to resolve [5].Then again, patients with “mild” Covid-19 may still complain about persistent symptoms, even weeks after the onset of symptoms.

In a large sample of hospitalized and non-hospitalized patients with confirmed or suspected Covid19, a list of 29 symptoms was completed. Fatigue and dyspnea were still very common, also in non-hospitalized patients [6].

Left atrial (LA) function is essential for optimal cardiac performance and regulates left ventricular (LV) filling [7]. Increasing interest in atrial size and function has shed light on the effects of atrial contribution on cardiovascular performance [8].

Sequelae to Covid-19 remain unexplored. Monitoring recovered patients is pivotal, as it is unclear whether the increased New York Heart Association (NYHA)-class will improve over time or not. Also, the effect of Covid-19 on LA functions has not been clearly studied [9]. Therefore, extended multidisciplinary indices, including clinical, laboratory tests, and left atrial function are required [10].

We aimed at the assessment of functional outcomes in post Covid-19 patients with persistent dyspnea using a multidisciplinary approach including clinical assessment, laboratory investigations, exercise ECG, and LA functions.

## Patients and methods

The present prospective, single-center, observational cross-sectional cohort study was conducted between May 2021 and February 2022 at cardiology out-patient clinic, Al-Zahraa University Hospital. The study included 60 post Covid-19 patients one month after recovery who suffered from persistent dyspnea with preserved LV systolic function compared to age- and sex- matched 30 healthy individuals as control group (with no history of previous Covid infection confirmed by negative reverse transcription polymerase chain reaction on a nasopharyngeal swab). Patients with hypertension, DM, smoking, known cardiomyopathies, and/or LV systolic dysfunction, significant valvular heart diseases, chronic lung disease, hypoxia, morbid obesity, chronic hepatic or renal disease, or contraindications to stress ECG were excluded from the study.

## Methodology

All studied cases were subjected to careful evaluation of dyspnea using different functional assessment scores and scales to determine the grade of dyspnea, including NYHA classification [11], modified Medical Research Council (mMRC) dyspnea scale [12] and Post-Covid-19 Functional Status scale (PCFS) [13]. Laboratory investigations included a complete blood picture (to determine WBCs, neutrophil/ lymphocyte ratio, ESR, and CRP), O2 saturation. CT chest was done for all patients during active Covid-19 infection five days after beginning of Covid symptoms and repeated before enrollment to the study to exclude chest involvement.

Tread-mill exercise testing was performed one month after recovery from Covid-19 using the standard Bruce protocol using GE machine. Maximum predicted heart rate (MPHR), blood pressure, heart rate, and exercise workload in metabolic equivalents (METs) were recorded. In addition, heart rate recovery (HRR) one minute and two-minutes were recorded with a cutoff value of ≤ 12 bpm was considered abnormal for 1-minute HRR [14] and ≤ 22 bpm was considered abnormal for 2-minutes HRR [15].

Conventional transthoracic echo-Doppler study was performed for all cases using Vivid-6 GE system (GE Ultrasound; Horten, Norway). Offline analysis was done using EchoPAC.GE version 110. All parameters were taken according to the basis of the American Society of Echocardiography [16]. LV dimensions, volumes, ejection fraction (EF), E and A velocities, E/A ratio. Tissue Doppler Imaging (TDI) for assessment of mitral annular systolic velocities “Sʼ”, early mitral annular diastolic velocities “Eʼ” and late mitral annular diastolic velocities “Aʼ” at the four annuli (septal, lateral, inferior, and anterior) were measured and an averaged Sa was obtained [17] in addition to (E/Eʼ ratio) [18].

LA dimensions, LA volumes, and LA volume index (LAVI) were calculated with LAVI cutoff value of 34 ml/m^2^ [19].

**Fig. 1:**
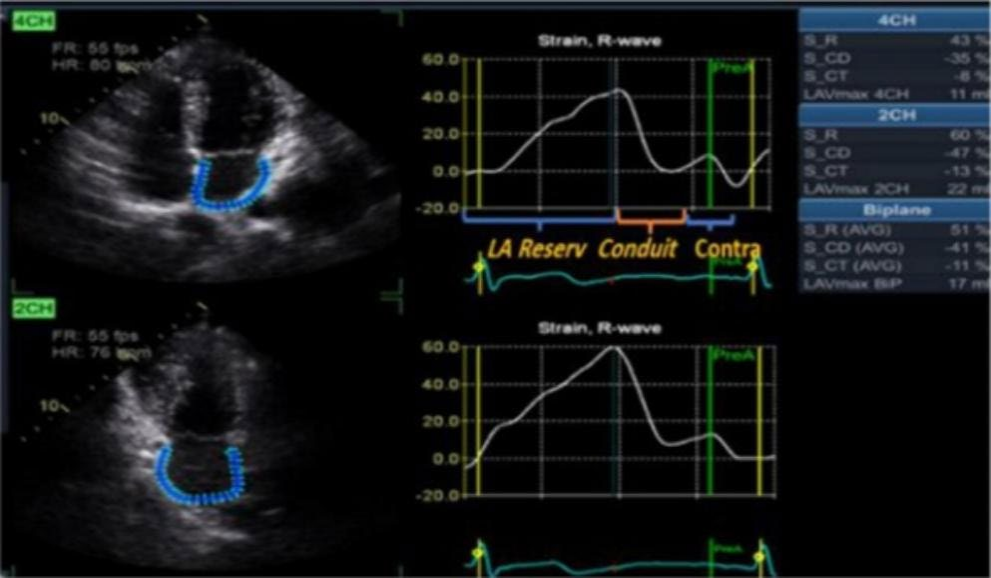
2D-speckle tracking of LA measuring reservoir, conduit and contractile functions

LA strain using 2D speckle tracking echocardiography was assessed in both the four-chamber, two-chamber views with manual correction if needed (Fig. 1). LA functions (reservoir = εS, conduit = εE, and contractile = εCT) were calculated automatically by the software of the left atrium using LA strain software (EchoPAC version 107) [20]. The normal reference ranges for reservoir strain, conduit strain, and contractile strain were 39%, 23%, and 17%, respectively [21].

Data on Covid-19 in Egypt were gathered from the websites of the WHO and the Egyptian Ministry of Health and Population, and the required sample size was calculated using a confidence interval of 85% and a margin of error of 10%. There were approximately 60 participants.

### Statistical analysis

The normality of distribution of all metric data was tested with the Shapiro–Wilk test. Parametric variables data are expressed as means with standard deviations. For the estimation of continuous variables, Student’s *t*-tests were used. Univariate analyses, chi-square analysis, Student’s unpaired *t*-test were used to compare variables between groups. To assess the association between variables, Pearson and Spearman correlation analyses were used. A multiple linear regression analysis for prediction of functional status was performed. *P*-values < 0.05 were considered statistically significant. The SPSS 23.0 software (SPSS Inc., Chicago, IL, USA) was used to measure all statistics.

## Results

The mean post Covid duration was 3.0 ± 1.7 months. There was significant impairment of functional status among post Covid patients compared to the control group as evidenced by higher classes at NYHA functional assessment, mMRC dyspnea scale and PCFS scale. No significant differences were found between two groups regarding weight, height, body surface area, or body mass index (Table 1).

**Table 1:** Comparison between post Covid patients and comtrol group regarding demographic data and functional status scores

Variables (mean ± SD)	Post-Covid patients N = 60	Control group N = 30	p value
**Age (years)**	33.6 ± 7.4	31.3 ± 8.4	0.167
**Sex**:			
• **Female**	53 (88.3%)	26 (86.7%)	NS
• **Male**	7 (11.7%)	4 (13.3%)	
**Post Covid duration (months)**	3.0 ± 1.7	-	-
**Palpitation**	39 (65%)	-	-
**Atypical chest pain**	32 (53.3%)	-	-
**Hospital admission during active Covid infection**	6 (10%)	-	-
**Home isolation during active Covid infection**	54 (90%)	-	-
**Results of CT during acute stage**			
• **Normal**	31 (51.7%)		
• **Abnormal**	29 (48.3%)	-	-
**Weight (Kg)**	73.6 ± 11.4	72.4 ± 9.1	0.578
**Height (cm)**	163.7 ± 4.4	164.2 ± 6.0	0.686
**BSA (m** ^**2**^ **)**	1.8 ± 0.2	1.8 ± 0.1	0.393
**BMI (Kg/m** ^**2**^ **)**	27.4 ± 3.9	26.3 ± 3.1	0.164
**NYHA class**	2.6 ± 0.5	0 ± 0	0.0001
**mMRC**	2.4 ± 0.7	0 ± 0	0.0001
**PCFS**	2.5 ± 0.7	0 ± 0	0.0001

Abbreviation: BSA = body surface area, BMI = mody mass index, NYHA = New York Heart.

Association, mMRC = modified Medical Research Council, PCFS = post Covid Functional status.

We found significant elevation of N/L ratio, ESR, and CRP and significant decrease of total leukocytic count of WBCs, neutrophils, lymphocytes, and platelets in post Covid patients compared to controls (Table 2).

**Table 2:** Comparison between post Covid patients and control group regarding laboratory investigations and tread-mill stress test results

Variables (mean ± SD)	Post-Covid patients N = 60	Control group N = 30	p value
**WBCs (x10³/cmm)**	3.0 ± 1.1	6.6 ± 1.6	0.0001
**Neutrophil(x10³/cmm)**	2.8 ± 1.2	3.6 ± 1.1	0.01
**Lymphocytes(x10³/cmm)**	1.1 ± 0.8	3.1 ± 0.9	0.0001
** N/L ratio**	2.6 ± 1.3	1.2 ± 0.4	0.0001
**Platelets (x10³/cmm)**	241.4 ± 54.7	266.4 ± 50.3	0.03
**HB%**	12.2 ± 1.0	12.3 ± 1.1	0.539
**ESR**	42.8 ± 16.2	10.1 ± 3.8	0.0001
**CRP (mg/L)**	7.8 ± 2.1	2.1 ± 0.9	0.0001
**Exercise duration (min)**	6.1 ± 1.3	9.8 ± 1.5	0.0001
**MPHR (bpm)**	159.6 ± 15.0	173.3 ± 14.1	0.0001
**%MPHR**	83.2 ± 6.5	90.2 ± 5.8	0.0001
**METs**	4.9 ± 0.9	7.9 ± 1.4	0.0001
**One-minute HRR (bpm)**	129.9 ± 9.5	103.3 ± 6.6	0.0001
**Two-minutes HRR (bpm)**	118.0 ± 8.1	84.2 ± 6.2	0.0001

Abbreviations: WBCs = while blood cell, N/L = neutrophil to lymphocyte ratio, HB = hemoglobin, ESR = erythrocytic sedimentation rate, CRP = C-reactive protein, MPHR = maximum predicted heart rate, METs = metabolic equivalents, HRR = heart rate recovery.

Post Covid patients showed a significant decrease in average exercise duration, average MPHR, %MPHR, and METs compared to the control group, while there were significant increase in one-minute HRR and two-minutes HRR in post Covid patients compared to the control group (Table 2).

Post Covid patients had a significant decrease of Av-Sa compared to the control group, but the value of Av-Sa in both groups was within normal cutoff value as mentioned in the methodology section. On the other hand, we could not find significant differences between both groups regarding LV dimensions, EF, and LV volumes. Post Covid patients showed a significant decrease of E and significantly higher ratio of LV E/E**ʼ** compared to control group. Post Covid patients showed significant increases in LA diameters, and LAVI compared to the control group. LA phasic functions evaluated by 2D STE (reservoir, conduit, and contractile) functions were significantly reduced in post Covid patients compared to the control group (Table 3).

**Table 3:** Comparison between post Covid patients and control group regarding LV and LA dimensions and functions

Variables (mean ± SD)	Post-Covid patients N = 60	Control group N = 30	p value
**LVEDD (mm)**	47.1 ± 4.9	47.0 ± 3.1	0.962
**LVESD (mm)**	29.4 ± 4.2	28.5 ± 3.1	0.303
**IVSd (mm)**	8.3 ± 0.8	8.4 ± 1.2	0.499
**LVPWd (mm)**	8.3 ± 0.9	8.5 ± 1.0	0.274
**EF (%)**	67.1 ± 5.4	68.0 ± 5.8	0.448
**LVEDV (ml)**	88.5 ± 26.8	85.4 ± 21.2	0.577
**LVESV (ml)**	33.5 ± 14.7	31.0 ± 7.6	0.374
**EF-biplane (%)**	62.9 ± 3.3	63.7 ± 4.0	0.11
**Average LV S-**_**4TDI**_ **(cm/Sect. )**	7.1 ± 1.2	7.3 ± 1.4	0.324
**LV E.vel (m/Sect. )**	79.6 ± 17.2	61.0 ± 13.4	0.0001
**LV A.vel (m/Sect. )**	50.2 ± 13.3	57.1 ± 18.5	0.366
**LV E/A**	1.6 ± 0.4	1.1 ± 0.3	0.557
**LV E/Eʼ**	9.6 ± 2.8	5.9 ± 1.3	0.0001
**LA ant-post (cm)**	30.3 ± 3.1	24.2 ± 3.9	0.0001
**LA sup-inf (cm)**	37.0 ± 2.6	27.6 ± 3.0	0.0001
**LA med-lat (cm)**	32.8 ± 3.4	29.4 ± 3.8	0.0001
**LAVI (ml/m** ^**2**^ **)**	34.1 ± 3.2	28.1 ± 1.9	0.0001
**Mean contractile strain (%)**	14.8 ± 3.9	21.7 ± 6.3	0.0001
**Mean conduit strain (%)**	20.4 ± 6.9	31.0 ± 6.1	0.0001
**Mean reservoir strain (%)**	37.3 ± 3.6	49.4 ± 6.8	0.0001

Abbreviations: LVEDD = LV end diastolic dimensions, LVESD = LV end systolic dimensions, IVSd = interventricular septum in diastole, LVPWd = LV posterior wall in diastole, LVEDV = LV end diastolic volume, LVESV = LV end systolic volume, LV S_− 4TDI=_average systolic 4-sites mitral annular velocities by tissue Doppler imaging, LV E/Eʼ=ratio of early diastolic mitral valve velocity/average early diastolic mitral annular velocities by TDI, Abbreviations: LAVI = left atrium volume index.

*Correlations between LA strain (reservoir, conduit and contractile) and different parameters*:

There were significant negative correlations between LA strain with NYHA class, mMRC scale and PCFS (except LA reservoir did not correlate significantly with PCFS). Also, LA strain correlated negatively with ESR, CRP and N/L ratio. Meanwhile, there were significant positive correlations between LA strain with exercise duration, METs, and MAPSE but correlated negatively with LAVI (Table 4).

**Table 4:** Correlations between LA functions and different parameters

Variables	LA reservoir strain		LA conduit strain		LA contractile strain	
	r	p	r	p	r	p
**NYHA**	-0.72	0.0001	-0.749	0.0001	-0.662	0.0001
**mMRC**	-0.679	0.0001	-0.754	0.0001	-0.635	0.0001
**PCFS**	-0.014	0.910	-0.750	0.0001	-0.527	0.0001
** N/L ratio**	-0.42	0.0001	-0.259	0.01	-0.235	0.02
**ESR**	-0.607	0.0001	-0.481	0.0001	-0.447	0.0001
**CRP**	-0.596	0.0001	-0.554	0.0001	-0.445	0.0001
**Exercise duration**	0.616	0.0001	0.625	0.0001	0.584	0.0001
**METs**	0.693	0.0001	0.686	0.0001	0.598	0.0001
**LAVI**	-0.526	0.0001	-0.735	0.0001	-0.62	0.0001
**MAPSE**	0.423	0.0001	0.317	0.0001	0.385	0.0001

Performing multiple linear regression analysis showed that LAVI (p < 0.001, 95%CI = 0.025 to 0.065), LA contractile strain (p < 0.001, 95% CI = − 0.035 to − 0.017) and LA reservoir strain (p < 0.003, 95%CI = − 0.018 to − 0.004) were independent predictors of the functional status in post Covid patients.

## Discussion

PASC (post-acute sequelae of Covid-19 infection) is a complex illness with widely varied manifestations that lacks a commonly accepted definition. PASC patients have symptoms that appear after Covid-19 infection and last for 4–12 weeks or more [22], and [23]. PASC can potentially cause unexplainable and persistent dyspnea (in the absence of cardiopulmonary problems).

The main findings of the current study were: (1) patients with post Covid dyspnea had persistent elevations of biomarkers and inflammation along with impaired functional capacity; (2) post-Covid patients demonstrated impaired LA strain (reservoir, conduit, and contractile) as well as LV diastolic dysfunction, and (3) impaired LA strain indices were found to be closely related to different functional scores, exercise duration, and METs in post Covid patients, implying that these are possible causes for post Covid dyspnea persistence.

Our study included 12.9% male patients and 89.1% female patients, suggesting a female preponderance among the studied post Covid population. Furthermore, Whitaker et al., 2021 [24] who conducted the REACT-2 (Real-time Assessment of Community Transmission Program), included over 500,000 people and identified female sex as a risk factor for long-term COVID symptoms (63.4%), implying that post-COVID persistent dyspnea is more common in the female population. ***Ganesh et al.*** [25] reported that 82% of post Covid patients were women, consistent with the known female predominance in PASC. An explanation for the role of hormonal dysregulation in females has been proposed, but PASC can also be the result of autoimmunity [26].

Females exhibit a more robust and enhanced CD8 T-cell activation response in acute Covid-19 infection, as well as decreased cytokine and monocyte release [27] and [28]. High levels of specific cytokines (TRAIL, IL-15) are linked to a worse clinical Covid-19 result in women [27]. These findings suggest an immune-mediated basis for the different course of the disease based on gender [27], and it is possible that these characteristics -the potent CD8 T-cell response, as well as the activation of Toll-like receptors TLRs and the inflammasome [29], -are likely involved in the sex differences observed in post Covid syndrome.

In our study, post-Covid patients had significantly worse functional status than the control group, as measured by higher classes of NYHA functional assessment, mMRC dyspnea scale, and PCFS scale. In respect to demographic characteristics, there were no major similarities between the two groups.

***Machado et al.*** [30] observed that in a middle-aged group of subjects without comorbid conditions and with a diagnosis of mild Covid-19 (i.e., not hospitalized during the infection), the majority (85%) reported mild functional limitations during the course of Covid-19. In agreement with our results, ***Lam et al.*** [31] examined 165 post Covid-19 patients with persistent symptoms for at least 4 weeks following acute Covid-19. 36% of patients had a 6-minute walk test distance less than the lower limit of normal and were considered to have reduced exertional tolerance. They reported that the subjective mMRC dyspnea score was significantly higher for the reduced exertional tolerance group compared to those in the normal exertional tolerance group. These patients with reduced exertional tolerance also reported more functional impairment, as reflected by a higher PCFS and a lower health-related quality of life score.

In the current study, post Covid patients demonstrated a significant increase of N/L ratio, ESR, and CRP with a significant decrease of the total leukocytic count of WBCs, neutrophils,, and platelets compared to control group. Our results were in agreement with those of ***Sonnweber et al.*** [32] who reported that there was a persistent elevation of CRP in post Covid patients. ***Evans et al.*** [33] examined 1170 individuals who were discharged from the hospital after receiving Covid-19 medication and discovered four groups of patients suffering from post-Covid syndrome. According to the investigators, CRP was especially high in the severe and very severe groups, probably due to post-Covid-19 systemic inflammation. Moreover, ***Ganesh et al.*** [25] demonstrated an elevated level of ESR and CRP 4-weeks post Covid infection with no significant sex difference.

Our study revealed that post Covid patients showed a significant decrease in average exercise duration, average MPHR, %MPHR, and METs compared to the control group, while there was a significant increase in one-minute HRR and two-minutes HRR in post Covid patients compared to the control group.

Our results were partially consistent with those of ***Mohr et al.*** [34] who included 10 post Covid-19 patients suffering from dyspnea who were evaluated by cardiopulmonary exercise testing (CPET). Mean heart rate during exercise was 133 ± 19 bpm (78.1 ± 7.3% for % MPHR). Mean value of lactate post exercise was 5.6 ± 1.8 mmol/l. The gap between reached peak work rate (92.4% predicted) to peak oxygen. In the study population, the disparity between reached max work rate (92.4% expected) and peak oxygen uptake (72.3% expected) might be explained by an early shift to anaerobic metabolism. This would explain why the mean value of lactate following exercise in the research cohort was so high. In most patients, muscular deficit and hence metabolic restriction may have contributed to dyspnea. In Covid-19, complete clinical recovery may take longer [35]. However, the cause of the muscle deficit is unknown. It could be attributed to atrophy as a result of insufficient physical load (e.g., prolonged bed rest), significant polyneuropathy, or direct Covid-19 damage to muscle or the central nervous system [36]. Despite the study conducted by [34] using CPET, that differs from our study in that we used the treadmill stress test in the evaluation of dyspnea in post Covid patients. Multiple hypothesis, including muscular deficiency and metabolic limitation, could explain the persistence of dyspnea in such patients.

Contrarily, ***Alba et al.*** [37] enrolled 18 patients with post Covid syndrome and matched comparator cohorts. Post Covid patients shared similar subjective dyspnea at referral (mMRC score; 1.6 ± 0.9 vs. 1.4 ± 0.9, p = 0.5). Post Covid patients had higher resting HR (77.5 vs. 73.5 bpm, p = 0.05), peak HR (162 vs. 138 bpm, p < 0.02) and MPHR% (91 vs.84%, p < 0.04). However, one- minute HRR (18.2 ± 8.3 vs. 21.2 ± 10 bpm; p = 0.4) and METs (5.4 vs. 5.6; p = 1.0) did not differ between both groups. They concluded that dyspnea in post Covid patients was due to heterogeneous pathophysiology.

Heart rate recovery (HRR) has been proposed to be affected by the interaction of variables such as exercise intensity [38], cardiac autonomic regulation, and level of physical fitness [39]. Several studies have also shown that exercise tolerance capacity is directly proportionate to HRR [40]. The previous data from different studies could explain the possible mechanisms of increased HRR and impaired exercise tolerance among our studied population.

In the current study, no significant differences were detected between both groups regarding LV dimensions, volumes, or EF. Meanwhile, post Covid patients showed a significant decrease of mitral valve E velocity and a significantly higher ratio of LV E/Eʼ (denoting diastolic dysfunction) compared to the control group. In addition, post Covid patients showed significant increases in LA diameters and LAVI compared to the control group. LA phasic functions evaluated by 2D STE, including reservoir, conduit, and contractile functions were significantly reduced in post Covid patients compared to the control group.

***Ingul et al.***, [41] enrolled 204 patients with Covid-19 (3 months after hospitalization) and 204 controls. In comparison to control group, post Covid patients had lower LVEF, LAVI, and Eʼ velocity (despite the fact that LVEF and LAVI did not show a clinical difference). However, there was no difference in Sʼ velocity or MV E/A ratio between the two groups. Most of the patients suffered from mild diastolic dysfunction.

Few researches evaluated the LA strain for prediction of atrial fibrillation (AF) in Covid patients. ***Beyls et al.***, [42] studied 79 Covid patients (16 patients had AF and 63 patients did not develop AF). A sub-analysis of Covid patients who did not develop AF revealed normal LVEF and volumes, as well as mild LV diastolic dysfunction (evidenced by E/Eʼ=8.5). In addition, LA volume (49 ml) was increased while LAVI was in the normal range (23 ml/m2). In comparison to the cutoff value specified in the methods section, LA strain measurements were reduced for reservoir (30.5%), conduit (17.2%), and contractile (13.3%) functions. LA strain analysis, particularly LA conduit strain analysis, has lately emerged as a powerful method for assessing LV diastolic dysfunction [43]. Covid-19 infection can result in myocardial diastolic dysfunction [44] through direct virus-related myocardial damage, inflammation, or cardiac fibrosis [45]. Covid-19 has the potential to set up subclinical LA dysfunction or exacerbate established LA dysfunction [46].

Among post Covid patients, LA strain (reservoir, conduit, and contractile) correlated negatively with NYHA class, mMRC scale and PCFS (except LA reservoir did not correlate significantly with PCFS). Also, a negative correlation was detected between LA strain and ESR, CRP, and N/L ratio. A significant positive correlation was demonstrated between LA strain with exercise duration, METs and MAPSE on the other hand, LA strain correlated negatively with LAVI.

Our results were concordant in the aspect of correlation between functional capacity and LA-phasic function, with results reported from ***Kusunose et al.*** [47] who studied the association of LA function with exercise capacity in patients with preserved ejection fraction. They found a reduction in LA reservoir function assessed by 2D STE in patients complaining of dyspnea and with reduced functional capacity. Also, they reported a significant positive correlation between METs and the LA volume, the E/Eʼ. However, their studied populations were different from our studied patients in regards to the age, gender, risk factors for cardiac disease, and history of Covid 19 infection.

**Ergül et al.** [9] reported that patients recovered from Covid-19 infection had increased LA maximum volume, LA pre-A volume, LA active EF, and a decrease in LA passive EF. **Barman et al.** [48] found higher LA diameters in patients with severe Covid-19. **Ergül et al.** [9] conducted the study on patients who recovered from Covid-19 and revealed that LA functions continued to deteriorate after the disease process. The worsening in LA functions observed in Covid patients could be attributed to systolic and diastolic dysfunction that arises during systemic inflammation.

The reason for diminished LA functions in patients who recovered from Covid-19 infection is unclear. Cytokine-mediated myocardial damage, oxygen supply–demand imbalance, microvascular and macrovascular thrombosis, endothelial damage, and direct viral invasion of the myocardium seem to play a role [49] and [50]. Systemic inflammation in Covid-19 can cause atrial myopathy, resulting in atrial arrhythmia. Atrial tissue stiffness is a fundamentally different risk factor and a potential cause of AF [51]. Endothelial dysfunction may play a crucial role in LA remodeling in patients recovered from Covid-19 and may cause LA dysfunction independent of ventricular function. Chronic LA remodeling is the final step in pressure overload that results in LA dilatation [52]. LA dilatation may be a manifestation of prolonged LV diastolic dysfunction or direct endothelial injury from SARS-CoV-2.

## Limitations

The first limitation of our study was the limited sample size. Only 10% of studied cases had a history of hospital admission with a moderate to severe form of Covid infection, which might have led to a selection bias. We suggest implementation of cardiopulmonary exercise testing rather than a tread-mill stress test for better evaluation of the patient’s symptoms and peak VO2. To support our findings, additional clinical trials with larger sample sizes and several CRP level tests, particularly at various post Covid periods, needed to be conducted. We were faced with the known limitations of LA strain analysis (far field, pulmonary veins, LA appendage orifice, and LA thin walls). LA strain analysis remains very sensitive for identifying LA functional changes in clinical practice. Finally, we did not include the vaccination status during the history taking of the included patients.

## Conclusion

Post Covid patients with persistent dyspnea exhibited persistently elevated inflammatory biomarkers as well as reduced functional capacity evaluated by different scores, impaired LA strain in addition to LV diastolic dysfunction. Further, impaired LA strain indices were closely correlated to functional scores, inflammatory biomarkers, exercise duration, and METs among post Covid patients, suggesting that these might be the possible etiologies for the persistence of post Covid dyspnea. Indexed LA volume and LA strain (reservoir and contractile) could predict the impairment of functional status in post Covid patients.
